# COVID-19 pandemic, lockdown, and consequences for a fossil fuel-dominated electricity system

**DOI:** 10.1063/5.0050551

**Published:** 2021-05-05

**Authors:** Imran Khan, Md. Sahabuddin

**Affiliations:** 1 Department of Electrical and Electronic Engineering, Jashore University of Science and Technology, Jashore 7408, Bangladesh; 2 Department of Biomedical Engineering, Jashore University of Science and Technology, Jashore 7408, Bangladesh

## Abstract

In South Asian countries, the spread of COVID-19 was not treated seriously until mid-March 2020. Measures similar to those considered in Europe and other developed countries, such as maintaining social distance and lockdowns, were imposed. Lockdowns imposed a significant impact on the power sector, and this has been well explored in the literature for developed countries. A country-specific assessment of the impact of COVID-19 on the energy sector is crucial for future crisis management and underpinning sustainable power sector development plans. The impact of COVID-19 on Bangladesh’s fossil-fuel dominated electricity sector is explored in this study. The analyses were conducted for 2019 and for the pandemic lockdown period in 2020. Daily hourly demand variations for different electricity generation zones in the country were investigated. The impact of these demand variations on greenhouse gas (GHG) emissions was assessed through time-varying carbon intensity analysis. Nationwide, the analysis revealed that the maximum hourly demand reduced by about 14% between 5 and 6 pm whereas the minimum demand reduction (3%–4%) occurred between 7:30 and 8 pm. Peak time demand reduction was found to be minimal during lockdowns. The national absolute GHG emission reduced by about 1075 kt CO_2_*e*, an ∼16% reduction compared with that in 2019. Time-varying carbon intensity patterns varied significantly between zones.

NOMENCLATURE

$CI\left(t\right)$: carbon intensity at a particular time period t
*EF*_*FuelGHG*_: fuel specific emission factor for a particular GHG
$E_{FuelGHG}\left(t\right)$: fuel specific emission for a particular GHG for a certain time period t
$E_{Fuel}^{CO_{2}-e}\left(t\right)$: fuel specific total carbon dioxide equivalent GHG emission at a particular time period t
$E_{FuelGHG}^{CO_{2}-e}\left(t\right)$: fuel specific carbon dioxide equivalent emission for that particular GHG for a certain time period t
$E_{Total}^{CO_{2}-e}\left(t\right)$: total carbon dioxide equivalent emissions from all fossil fuels and all GHGs at a particular time period t
*GEF*_*FuelGHG*_: fuel specific generation emission factor for a particular GHG
*G*_*Fuel*_(*t*): fuel specific electricity generation in kWh for a time period t
*G*_*Fuel*_(*t*): fuel specific electricity generation at a particular time period t
$G_{Total}\left(t\right)$: total system generation from all fuels (including renewables and non-renewables) at a particular time period t
*GWP*_*GHG*_: global warming potential of different GHGs
*η*_*Fuel*_: fuel specific power plant’s efficiency

## I. INTRODUCTION

Energy, particularly the use of electricity, is strongly related to the economic activity of any country. However, due to the COVID-19 pandemic, there will be short- and long-term impact on the electricity sector globally. For instance, the global electricity demand decreased by about 15% due to the pandemic (Chiaramonti and Maniatis, 2020). This impact included not only economic and energy-related issues but also environmental factors such as greenhouse gas (GHG) emissions from electricity generation (Khan, 2019d). For instance, the International Energy Agency reported that primary energy demand and global energy-related CO_2_ emissions reduced by 4% and 5.8%, respectively, in 2020; the CO_2_ emission reduction was 3.3% in the power sector in 2020 (IEA, 2021).

Recent studies have focused on many different aspects of the COVID-19 pandemic and energy systems. For example, a significant impact on energy demand was observed in China (Norouzi *et al*., 2020). The impact on household energy use in China before, during, and after lockdowns due to the COVID-19 pandemic was also explored (Cheshmehzangi, 2020). Another recent study investigated the overall environmental impact of lockdowns, predominantly in the South Asia region (Arora *et al*., 2020). The authors found a significant reduction in air and noise pollution. However, the study did not pay particular attention to the electricity sector’s impact on the environment. A similar environmental assessment was also conducted in China (Wang and Su, 2020). The COVID-19 pandemic and its impact on India’s economy were assessed by observing electricity consumption and nighttime light intensity (Beyer *et al*., 2021).

Steffen *et al.* (2020) analyzed the policy responses that should be adopted to cope with the COVID-19 energy crisis, and the authors proposed three different time horizons to tackle this kind of pandemic situation in the energy sector: short-, mid-, and long-term plans. Short-term plans deal with immediate crisis response, mid-term plans focus on economic recovery in the energy sector through new opportunities, and long-term plans deal with making energy transitions a shock-proof sector through appropriate policy development.

One study reviewed the impact of COVID-19 from consumers’ point of view and critically analyzed many different measures taken by governments around the world (Mastropietro *et al*., 2020). For instance, the authors reported that “bill reductions and cancellations” should not be provided to all consumers as it is an inefficient approach. Similarly, a recent study reviewed the action plans of the G20 member countries regarding electricity consumption during the pandemic (Qarnain *et al*., 2020). For developing nations, such as African countries, preliminary responses to the pandemic in the energy sector were short-term schemes, including free electricity, VAT exemption, and waiving electricity bill payments (Akrofi and Antwi, 2020). Similar initiatives were also undertaken by the Government of Bangladesh. The government declared that “*Household consumers will be able to pay their delayed electricity bills for the months of February–June by July 31 without fines*” (Sajid, 2020a).

To identify the electricity consumption gap due to the COVID-19 pandemic, a prediction-based analysis method was proposed in Huang *et al*. (2021). A study in the USA found that “*Energy sovereignty is a critical component in the design of a post-COVID-19 energy system that is capable of being resilient to future shocks without exacerbating injustices that are killing the most vulnerable among us*” (Brosemer *et al*., 2020). A data-driven analysis in the USA found that both power demand and electricity prices were reduced during the pandemic (Ruan *et al*., 2021). The electricity generation from three major regional transmission organizations in the USA—the New York ISO, the Pennsylvania–New Jersey–Maryland Interconnection, and the Midcontinent ISO—was shifted from both base and peak load sources after the stay-at-home instructions were issued (Eryilmaz *et al*., 2020). Based on multivariate time series forecasting with bidirectional long- and short-term memory, the influence of the COVID-19 pandemic on electricity demand in the UK was investigated (Liu and Lin, 2021), and it was found that the electricity demand pattern followed the weekend’s demand profile during the lockdown. Using data visualization and descriptive statistics, the behavior of different European electricity systems including the German electricity system during the pandemic was identified in Halbrügge *et al*. (2021).

Closer to this study, Rugani and Caro (2020) assessed the impact of the COVID-19 pandemic on the environment in Italy in terms of direct and indirect GHG emissions from electricity consumption. The authors found that due to the lockdown, Italy was able to avoid 5.6 to 10.6 Mt CO_2_*e* for the period March–April 2020. Similarly, in ON, Canada, there was a reduction of about 1267 GW (14%) of electricity demand due to the pandemic in April 2020 (Abu-Rayash and Dincer, 2020). Consequently, CO_2_*e* declined by 40 000 tonnes, which is a positive impact on the environment for the same time period. In the USA, the electricity demand reduction was about 10%, and as a result, CO_2_ emissions declined by 15% for the lockdown period (Gillingham *et al*., 2020).

In the developing world, the impact of this pandemic is devastating and will hinder the development for achieving sustainable development goals by 2030. Goal-7 is one of the crucial sustainable development goals and is associated with “affordable and clean energy for all,” and developing countries’ energy sector impact assessment is an utmost priority for this pandemic situation. Immediate policy measures need to be initiated, as the one identified in a recent study: “*If sustainability is to be revived as a development objective, then low and middle-income economies will need to come up with policies that are affordable and achieve multiple SDGs simultaneously*” (Barbier and Burgess, 2020). Hence, the objective of this study is to analyze the impact of the COVID-19 pandemic on a fossil fuel-dominated electricity generation system in a least developed country, Bangladesh.

Previous studies have revealed electricity demand or consumption scenarios in developed economies. For instance, the cumulative decrease in electricity consumption in the EU countries and the states of the USA was between 3% and 12%, respectively, following the stay-home orders for five months (Prol and O, 2020). However, a recent review found that “*fully enforced lockdowns and stay home orders have increased the residential sector energy demand by a range from 11% to 32% for several countries*” (Krarti and Aldubyan, 2021). The electricity demand reduced by about 20% in Italy and France (Chiaramonti and Maniatis, 2020). In Greece, the demand reduction was about 1%–5%, whereas the change was minimal in Germany (EURELECTRIC, 2020). Electricity consumption in Austria and Denmark reduced within the range of 10%–15% (EURELECTRIC, 2020). Energy demand variations for different buildings such as residences, offices, and schools in a district in Sweden were assessed through simulation to identify the impact of the COVID-19 crisis (Zhang *et al*., 2020). In Spain, the consumption decreased by 13.49% between 14 March and 30 April 2020, compared to the last five years’ average for the same duration (Santiago *et al*., 2021). In contrast, another study in Spain found that residential consumers increased their consumption during the total lockdown and the reopening period by about 15% and 7.5%, respectively (García *et al*., 2021).

The global reduction in consumption during the total lockdown by non-residential consumers was about 38%, and during the reopening period, it was around 14.5% (García *et al*., 2021). In Australia, the impact of lockdowns due to the COVID-19 pandemic on energy use and peak demand in residential aged care (RAC) facilities was investigated, and it was found that the energy use and peak demand reduction pattern depends on the geographical location of the facility (Liu *et al*., 2021). For instance, the highest reduction in peak demand and energy use was observed in facilities that are in warm regions.

A recent study explored the spatial and temporal variations for different countries dominated by developed ones and found that the variations were complicated (Jiang *et al*., 2021). The same is true for a study in Lagos, Nigeria, in which the authors considered three different scenarios—business-as-usual, partial lockdowns, and total lockdowns—and assessed the changes in electricity demand and consumption (Edomah and Ndulue, 2020). However, the spatial and temporal variations of electricity demand, generation, and environmental impact within a developing country have not been explored well in the literature. The focus of this study is twofold: first, to check the impact of lockdowns on electricity demand and generation for Bangladesh and second, to check the consequences of this demand variation from an environmental perspective, more specifically carbon emission intensity (in short, carbon intensity).

### A. Electricity system in Bangladesh

The electricity generation system in Bangladesh is dominated by fossil fuel generation. According to the Bangladesh Power Development Board (BPDB) and the Sustainable and Renewable Energy Development Authority (SREDA), fossil fuel and renewable generation capacities are at about 20 548 MW and 647.49 MW, respectively (BPDB, 2020a and SREDA, 2020). These capacities’ breakdowns are shown in Tables I and II

**TABLE I.** Fossil fueled and imported electricity generation capacity of Bangladesh [data source: BPDB (2020a)] (HFO—heavy fuel oil and HSD— high-speed diesel).

Fuel type	Capacity (MW)
Coal	524
Gas	11 502
Oil (HFO and HSD)	7 362
Import from India	1 160
Total	20 548

**TABLE II.** Renewable electricity generation capacity of Bangladesh [data source: SREDA (2020)].

	On-grid	Off-grid	Total capacity
Fuel type	(MW)	(MW)	(MW)
Solar	87.05	326.51	413.56
Hydro	230	0	230
Wind	0.9	2	2.9
Biogas to electricity	0	0.63	0.63
Biomass to electricity	0	0.4	0.4
Total	317.95	329.54	647.49

**FIG. 1.**
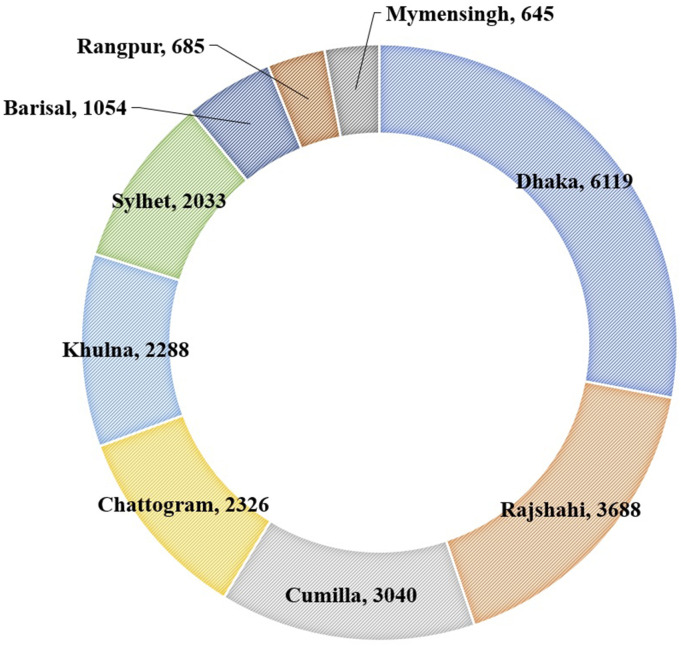
Zone-specific installed generation capacities in MW (as of 6 August 2020) [data source: BPDB (2020b)].

The BPDB has divided the country into nine electricity generation zones, as illustrated in Fig. 1. These generation zones include both renewable and non-renewable sources. For instance, 230 MW hydro and 27 MW solar generations (from power plants) are included in the Chattogram zone. The other solar generations were from 3 MW to 8 MW solar power plants in the Mymensingh and Rangpur zones. Although the Dhaka zone has the highest generation capacity (6119 MW), the actual derated capacity is 5895 MW. The derated capacities for Chattogram, Cumilla, Mymensingh, Sylhet, Khulna, Rajshahi, and Rangpur were found to be 2266, 2980, 637, 1988, 2284, 3668, and 605 MW, respectively. In terms of energy trading, 160 and 1000 MW electricity was imported from India through Cumilla and Khulna zones, respectively.

The total retail electricity consumption in Bangladesh was 63 364 GWh in the financial year 2019–2020, of which the dominant sector was residential, with a share of 57.02%, followed by industrial (27.58%), commercial (10.19%), and agriculture (2.42%); the rest were other sectors (2.79%) (BPDB, 2020c).

**FIG. 2.**
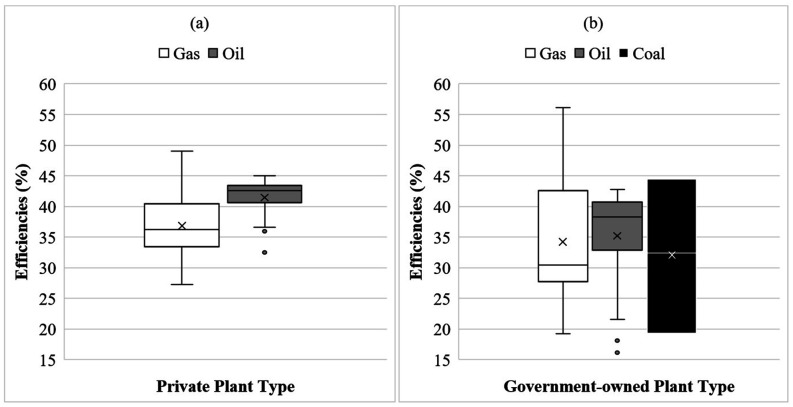
Box and whisker plot showing (a) private- and (b) government-owned power plants’ efficiencies in Bangladesh. The horizontal line and cross indicate the median and average value for that fuel type within each box, respectively. The lower (and upper) edges of the box are the 25th (75th) percentile. Whiskers represent the upper and lower ranges, and the dots represent the outliers [data source: BPDB (2019) and EQMS (2015)].

According to the BPDB’s annual report (BPDB, 2019), there are two types of power plant owners in Bangladesh: government and private. The power plants in the private sector are mainly gas- and oil-fired, and the efficiencies of these plants vary over a wide range. For example, the lowest efficiency for gas- and oil-based power plants were found to be 27.25 and 32.5, respectively. The highest efficiencies were 49.06 and 44.97, respectively. This is depicted in Fig. 2(a)

The lowest efficiencies of the government-owned power plants were found to be 19.28, 16.14, and 19.36 for gas-, oil-, and coal-fueled plants, whereas the maximum efficiencies were 56.13, 42.74, and 44.49 for the same types of plants, respectively. This is shown in Fig. 2(b). For the government-owned power plants, zone-specific average plant efficiencies varied between 30.27 and 40.80 for gas-fired ones and 19.81 and 40.32 for oil-fired ones. The average efficiency of the gas-fired power plants in the Cumilla zone was found to be higher than that in the other zones. For the oil-fired plants, the efficiency was found to be comparatively better in the Dhaka zone than in the others. Overall, the private sector’s power plant efficiencies were found to be better than those of the government-owned power plants in Bangladesh.

The rest of the article is organized as follows: Sec. II explains the data and method used for the analysis. Section III presents the results and related analysis. Section IV discusses the findings, and the final section concludes the article.

## II. DATA AND METHOD

For this analysis, daily, hourly, and half-hourly electricity generation data were collected from the Bangladesh Power Development Board (BPDB) and the Power Grid Company of Bangladesh (PGCB) websites (PGCB, 2020 and BPDB, 2020d). Data were considered from the end of March to the end of May of 2019 and 2020 so that a comparison of the lockdown and the baseline situation can be made. Many recent studies also considered these two consecutive years for this type of analysis; for example, see Mastropietro *et al.* (2020) and Abu-Rayash and Dincer (2020).

The time-varying carbon intensity approach is chosen for emission analysis as it is able to investigate a number of factors at one time, such as identifying temporal carbon intensity variation, determining peak carbon-intensive (carbon peak) hours, and identifying the dominant fuel contributing to emission reduction or acceleration; for further details, see Khan (2019d) and Khan *et al.* (2018). Previous studies considered average efficiencies of different electricity generation technologies, whereas in this study, plant-specific efficiencies were used. Thus, this analysis provides more accurate emission data from individual power plants in Bangladesh. Greenhouse gas emissions and time-varying carbon intensities were calculated using the following formulas [adopted from Khan (2018) and Khan (2019d)]:

$$GEF_{FuelGHG}=\frac{EF_{FuelGHG}}{\eta_{Fuel}}$$ (1)

$$E_{FuelGHG}\left(t\right)=G_{Fuel}\left(t\right)\times GEF_{FuelGHG}$$ (2)

$$E_{FuelGHG}^{CO_{2}-e}\left(t\right)=E_{FuelGHG}\left(t\right)\times GWP_{GHG}$$ (3)

*Here*, *GWP*_*GHG*_ = 1 *for* CO_2_, 25 *for* CH_4_, *and* 298 *for* N_2_O,

$$E_{Fuel}^{CO_{2}-e}\left(t\right)=\sum_{GHGs}E_{FuelGHG}^{CO_{2}-e}\left(t\right)$$ (4)

$$E_{Total}^{CO_{2}-e}\left(t\right)=\sum_{Fuels}E_{Fuel}^{CO_{2}-e}\left(t\right)$$ (5)

$$G_{Total}\left(t\right)=\sum_{Fuels}G_{Fuel}\left(t\right)$$ (6)

$$CI\left(t\right)=\frac{E_{Total}^{CO_{2}-e}\left(t\right)}{G_{Total}\left(t\right)}$$ (7)

For emission calculation, three major GHGs—carbon dioxide (CO_2_), methane (CH_4_), and nitrous oxide (N_2_O)—were considered for this analysis. As no national GHG inventory is available in Bangladesh, to calculate *GEF*_*FuelGHG*_, the fuel-specific emission factor for a particular fuel was considered from the Intergovernmental Panel on Climate Change (IPCC), Fourth Assessment Report (AR4) (IPCC, 2007). Although the IPCC Fifth Assessment Report (AR5) was available, the AR4 was used for this analysis so that the results can be compared with those of the previous literature for Bangladesh (Khan, 2018). These emission factors are listed in Table III. The coal used for electricity generation in Bangladesh is mainly sub-bituminous coal, and the characteristics of heavy fuel oil (HFO) and high-speed diesel (HSD) oils are in the distillate fuel oil No. 2 category; hence, these were considered for emission factor selection (cf. Table III). While life cycle emissions were present from renewable sources such as hydro and solar, they were assumed to be emission-free due to their insignificant contribution compared to fossil-fuel emissions. Fuel specific power plant efficiencies were taken into account from the BPDB annual report and the environmental impact assessment (EIA) report of specific power plants (cf. Fig. 2) (BPDB, 2019 and EQMS, 2015).

**TABLE III.** Fuel-specific emission factors [adopted from IPCC (2007)] (note: 1 mm BTU = 293.07 kWh).

	kgCO_2_ per	gCH_4_ per	gN_2_O per
Fuel	mm BTU	mm BTU	mm BTU
Sub-bituminous coal	97.17	11.00	1.60
Natural gas	53.06	1.00	0.10
Distillate fuel oil No. 2 (used for HFO and HSD)	73.96	3.00	0.60

## III. RESULTS AND ANALYSIS

The analysis was conducted according to the BPDB generation zones. Diverse changes in electricity demand and emissions were observed in these zones.

**FIG. 3.**
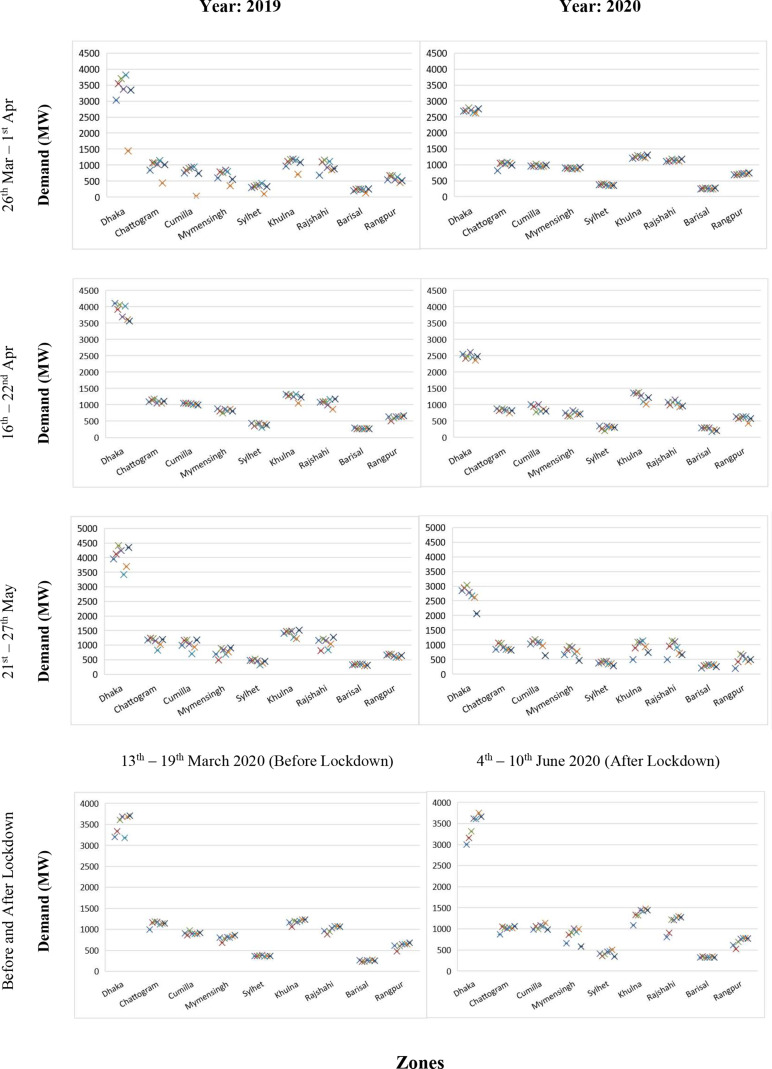
Electricity daily demand variations at different zones in Bangladesh before, during, and after the lockdown period (for interpretation of the references to color in this figure legend, the reader is referred to the Web version of this article).

**FIG. 4.**
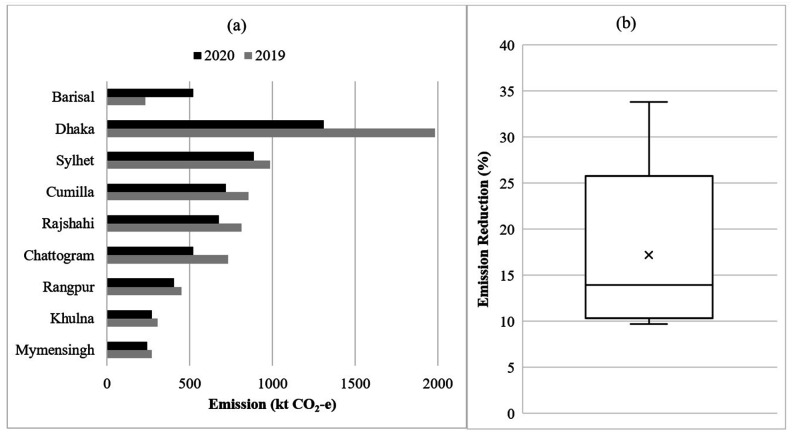
(a) Zonal emission for the lockdown period (23 March–30 May) in 2020 and emission in 2019 for the same period, (b) the box and whisker plot showing the percentage of emission reduction in 2020 compared with 2019 during the lockdown period in Bangladesh for eight different zones (Barisal was excluded as emission increased during the same period). The horizontal line and cross indicate the median and average value of carbon intensities within each box, respectively. The lower (and upper) edges of the box are the 25th (75th) percentile. Whiskers represent the upper and lower ranges.

### A. Electricity demand variations

To check the demand variations, three different weeks from three different months were taken into account during the lockdown period: 26 March–1 April, 16–22 April, and 21–27 May 2020. The same calendar days were also considered from 2019 for comparison purposes. Unfortunately, for 26 and 27 May 2019, demand data were missing in the online database; thus, demand data of 19–25 May 2019 were used for comparison. These daily demand variations are plotted in Fig. 3

During 26 March–1 April, the daily electricity demand in the Dhaka zone reduced significantly from the range 3000–4000 MW in 2019 to 2300–2800 MW in 2020. For the other zones, demand reduction was insignificant. Interestingly, the demand fluctuations on weekdays and weekends were completely absent during the lockdown period in 2020.

For the week 16–22 April 2020, the Dhaka zone’s demand reduced further and reached below 2500 MW. The demand in Chattogram was below 1000 MW in 2020, which was above 1000 MW in the previous year for the same calendar days. The demand also reduced for Cumilla. For the other zones, changes in demands were marginal. Nevertheless, daily demand fluctuations were found in this week during the lockdown period, perhaps due to residential demand variations.

Compared to the period April 21–27 May 2020, the demand in the Dhaka zone increased and varied between 2500 MW and 3000 MW, while that in Chattogram remained below 1000 MW for this week as well. For the Khulna zone, electricity demand reduced in 2020 compared to the demand in 2019 during the same week. For instance, the maximum demand was found to be 1508 MW in 2019 but was 1133 MW in 2020. Although fluctuations in demand were found for other zones in 2020, the demand range did not reduce significantly.

Before and after the lockdown, the Dhaka zone’s demand varied between 3003 and 3752 MW, whereas this variation was between 2354 and 2705 MW during the lockdown. Similarly, for Chattogram, the demand variations before and after lockdown were 991–1180 MW and 870–1059 MW, respectively; during the lockdown, this variation was between 821 and 1068 MW. Overall, substantial changes in electricity demand during the lockdown period were observed predominantly for Dhaka and Chattogram.

Note that as the comparisons were made during the same time period for both years, the impact of weather was not considered. However, it can be seen that the electricity demands in all the zones were higher in June than in March due to the increased temperature and humidity for the latter month (cf. Fig. 3).

### B. Carbon intensity variations

In terms of absolute emission, the analysis revealed that due to the lockdown, a decline of 1075 kt CO_2_*e* of GHG emissions was recorded compared to the previous year. Thus, a 16% emission reduction was observed compared with the previous year for the same time period. Zone-specific absolute emissions between the previous and this year show that emissions were reduced for every zone except Barisal. This is depicted in Fig. 4(a)

Emission reduction was observed in different zones, ranging from 9% to 33% [see Fig. 4(b)]. The average reduction percentage was found to be 17%. The highest reduction in GHG emission was found for the Dhaka zone, followed by Chattogram, Rajshahi, and Cumilla, and the minimum reduction was found for Rangpur. Barisal’s emission increased from 232 kt CO_2_*e* in 2019 to 520 kt CO_2_*e* in 2020, which is about a 124% increase. This is due to the fact that a coal-fired power plant with a present capacity of 622 MW (which will be expanded to 1320 MW) came online in January 2020. Although it uses ultra-supercritical power generating units, emissions increased more than double the figure for the previous year.

**FIG. 5.**
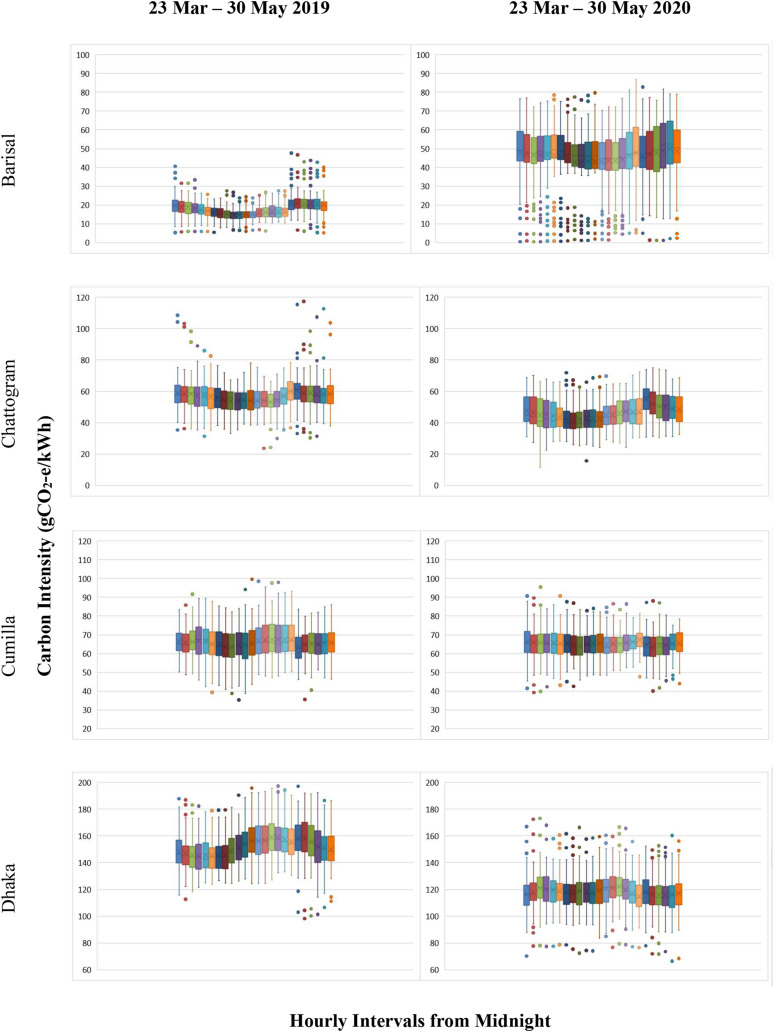
Box and whisker plot showing carbon intensity variations in Bangladesh for Barisal, Chattogram, Cumilla, and Dhaka between 23 March and 30 May for 2019 and 2020. The horizontal line and cross indicate the median and average value of carbon intensities within each box, respectively. The lower (and upper) edges of the box arethe 25th (75th) percentile. Whiskers represent the upper and lower ranges, and the dots represent the outliers. Note: The lockdown period due to the COVID-19 pandemicwas from 23 March to 30 May 2020 in Bangladesh (for interpretation of the references to color in this figure legend, the reader is referred to the Web version of this article).

The time-varying carbon intensity analysis for different zones provided detailed insights into the emission patterns on an hourly basis, and this varied substantially from one zone to another. For example, drastic changes in hourly emissions were observed for Barisal due to the addition of a coal-fired power plant in the generation fleet. Notably, the emissions at peak hours during the lockdown increased more than threefold compared to those in 2019 (see Fig. 5).

For Chattogram, the 24 h carbon intensity (CI) in 2019 was found to be almost flat, and the average hourly CI range was between 53 and 60 gCO_2_*e*/kWh. In contrast, the average hourly CI was found to be in the range 41–55 gCO_2_*e*/kWh during the lockdown period in 2020. Importantly, noticeable fluctuations were observed for the 24 h carbon intensity profiles, and the evening peak is clearly visible (see Fig. 5). The reason might be that residential electricity usage, as all other industrial and commercial activities, was suspended for the lockdown period.

In Cumilla, the carbon intensity varied between 38 and 95 gCO_2_*e*/kWh in 2019. In 2020, during the lockdown period, this range varied between 45 and 87 gCO_2_*e*/kWh. Noticeable reductions in carbon intensity were observed from 1 to 6 pm; CI reduced during the afternoon hours in Cumilla. The average CI was between 60 and 70 gCO_2_*e*/kWh for both years.

A dramatic reduction in CI was observed for Dhaka during the lockdown in 2020. For instance, the average hourly CI varied between 145 and 159 gCO_2_*e*/kWh in 2019. Contrarily, this range was 115–122 gCO_2_*e*/kWh during the lockdown in 2020. During the non-pandemic period, there were two CI peaks in Dhaka—one at 3 pm and another at 8 pm. Nevertheless, during the lockdown period, CI was almost flat all through the day (see Fig. 5).

**FIG. 6.**
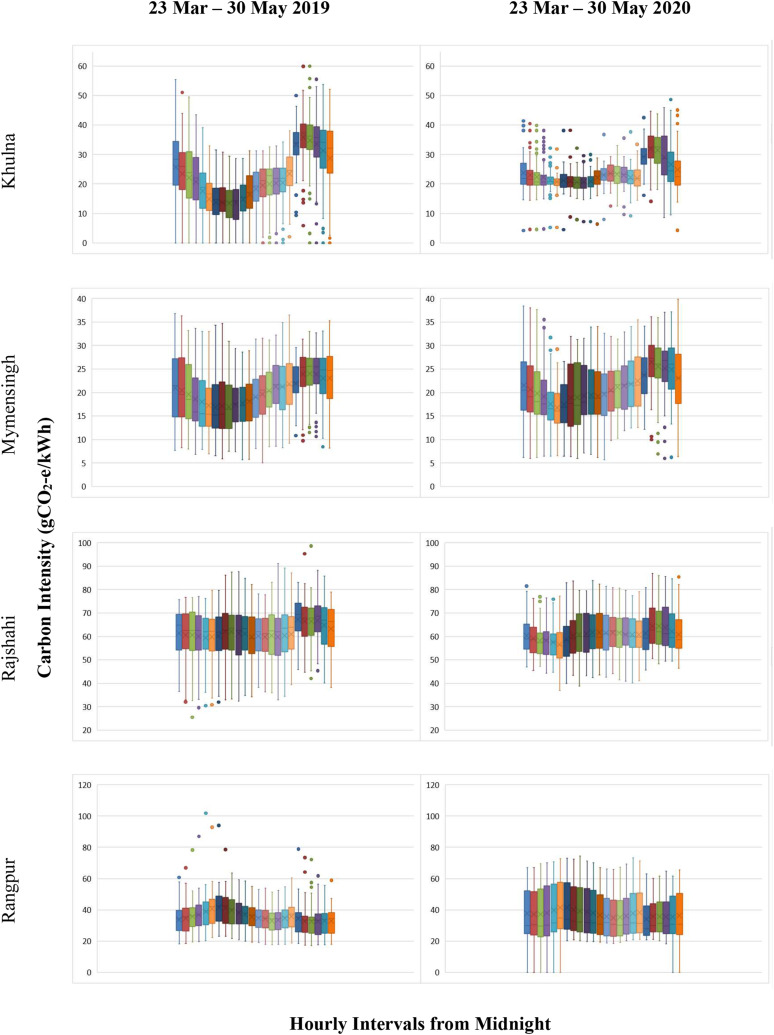
Box and whisker plot showing carbon intensity variations in Bangladesh for Khulna, Mymensingh, Rajshahi, and Rangpur between 23 March and 30 May for 2019 and 2020. The horizontal line and cross indicate the median and average value of carbon intensities within each box, respectively. The lower (and upper) edges of the box are the 25th (75th) percentile. Whiskers represent the upper and lower ranges, and the dots represent the outliers. Note: The lockdown period due to the COVID-19 pandemic was from 23 March to 30 May 2020 in Bangladesh (for interpretation of the references to color in this figure legend, the reader is referred to the Web version of this article).

During the non-pandemic situation in 2019, the lowest average CI (13.42 gCO_2_*e*/kWh) was found at 9 am, and the highest average was 35.81 gCO_2_*e*/kWh at 8 pm in Khulna. A sharp increase in CI can be seen during peak hours (5–11 pm) in 2019. One of the reasons for this sharp increase is that most of the power plants in this zone are oil-fired and serve as peaking power plants. Notably, oil is more carbon-intensive than natural gas. For the lockdown period in 2020, from 1 am to 6 pm, the average hourly CI became almost flat and varied between 20.72 and 23.82 gCO_2_*e*/kWh, whereas during peak hours, this range increased to 22–32 gCO_2_*e*/kWh. For both years, the whiskers and outliers represent the peaking power plants’ activity-related emissions (see Fig. 6).

In Mymensingh, the hourly CI change during the lockdown in 2020 was marginal compared to 2019. For example, a noticeable change was only observed between 5 and 7 am. Similarly, the hourly CI reduced from midnight to early morning for the Rajshahi zone during the lockdown in 2020 compared with 2019. Although the CI pattern in 2020 was almost identical to that in 2019 in Rangpur, hourly CI fluctuations were evident. For example, at 6 am, the CI fluctuated between 31.32 (25th percentile) and 46.79 gCO_2_*e*/kWh (75th percentile) in 2019, whereas this variation was 27.84–57.70 gCO_2_*e*/kWh in 2020 during the lockdown period. Interestingly, a carbon-intensive peak was observed in the morning for this zone (see Fig. 6).

**FIG. 7.**
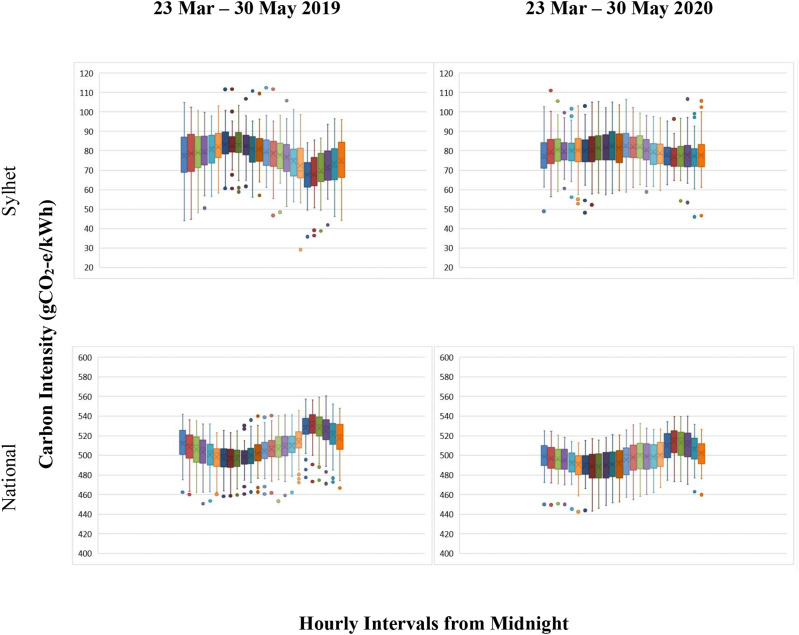
Box and whisker plot showing carbon intensity variations in Bangladesh for Sylhet and National between 23 March and 30 May for 2019 and 2020. The horizontal line and cross indicate the median and average value of carbon intensities within each box, respectively. The lower (and upper) edges of the box are the 25th (75th) percentile. Whiskers represent the upper and lower ranges, and the dots represent the outliers. Note: The lockdown period due to the COVID-19 pandemic was from 23 March to 30 May 2020 in Bangladesh (for interpretation of the references to color in this figure legend, the reader is referred to the Web version of this article).

It can be seen from Fig. 7 that Sylhet has a morning carbon peak (“carbon peak” and “carbon intensity peak” are used interchangeably in the text) during non-pandemic hours in 2019. In contrast to most other zones, Sylhet showed a drop in carbon intensity during evening peak hours. In 2020, the morning peak shifted to 11 am and created a day carbon peak because of the impact of lockdowns. On the other hand, an evening drop in carbon intensity was not found during the lockdown (see Fig. 7).

The national average hourly CI varied between 496 and 530 gCO_2_*e*/kWh in 2019. A clear carbon peak was also present at 8 pm. For the lockdown period in 2020, the average hourly CI range was found to be 488–514 gCO_2_*e*/kWh, with a similar carbon peak in the evening. Although hourly CI fluctuations were observed all day, the maximum fluctuations were found during the middle of the day (see Fig. 7).

In summary, time-varying carbon intensity analysis revealed the hourly emission status for different zones in Bangladesh. Of the nine zones, Cumilla and Dhaka had day carbon-intensive peaks; Barisal, Chattogram, Khulna, and Rajshahi had carbon-intensive peaks in the evening. Mymensingh has a midnight carbon peak whereas Rangpur and Sylhet showed carbon-intensive peaks in the morning for the non-pandemic period. During the lockdown in 2020, there was no day carbon-intensive peak found for Cumilla. Late night and afternoon carbon peaks were observed for Dhaka. No evening carbon peak was found for Barisal.

On the other hand, Chattogram, Khulna, Mymensingh, and Rajshahi had a peak in the evening. An early morning carbon peak was found for Rangpur. Interestingly, Sylhet’s morning carbon peak shifted from 7 to 11 am as a day peak. In terms of national carbon intensity, the overall pattern was identical to that of the non-lockdown situation. However, CI reduced during the lockdown. At the same time, hourly fluctuations increased during the lockdown period.

## IV. DISCUSSION

**FIG. 8.**
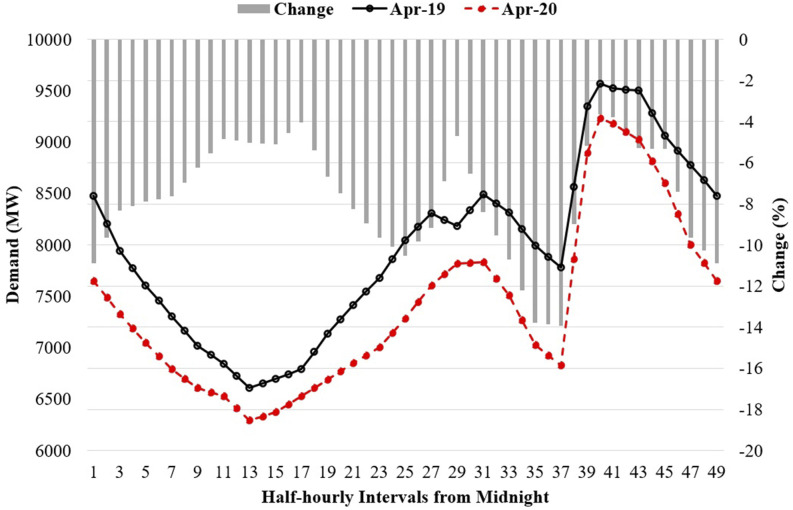
Three weeks’ averaged demand profiles for April in 2019 (solid line) and 2020 (dotted line) and change (%) in demand between these two years (for interpretation of the references to color in this figure legend, the reader is referred to the Web version of this article).

It is evident from the analysis results that daily demand variations for most of the zones in Bangladesh are not significant. For instance, a dramatic decrease in daily demand was found for Dhaka, whereas for many other zones such as Barisal and Sylhet, this reduction was marginal. Importantly, these zonal demand variations altogether have an impact on the national demand profile. For example, the average half-hourly demand profiles for three weeks—from 1 to 21 April for both 2019 and 2020—are plotted in Fig. 8, along with their changes.

Electricity demand reduced for the lockdown period in 2020 compared to 2019. The maximum reduction was about 14% in the afternoon, predominantly between 5 and 6 pm (see Fig. 8). On the other hand, during evening peak hours, this reduction was within the range of 3%–5%. During the day peak hours, the demand reduction was 4%–8%. Peak demand reduction due to the COVID-19 pandemic and the lockdown was also reported in ON, Canada (Abu-Rayash and Dincer, 2020). A similar situation was also observed in NY, USA (Chen *et al*., 2020).

In the industrial and commercial sectors, electricity consumption dropped by about 50% and 40%, respectively, due to this pandemic, whereas consumption increased by about 15% in the residential sector (FE, 2020). More than half (57.02%) of the electricity demand in Bangladesh is from the residential sector (BPDB, 2020c). This also justifies the minimum demand reduction during the network peak hours in Bangladesh. Domestic electricity consumption or demand also increased in other countries during the lockdown, and it ranges from 11% to 32% (Krarti and Aldubyan, 2021). For example, in Bulgaria, domestic electricity consumption increased by 1.18% (EURELECTRIC, 2020). In Spain, residential consumers increased their consumption by 15% during the lockdown (García *et al*., 2021).

**FIG. 9.**
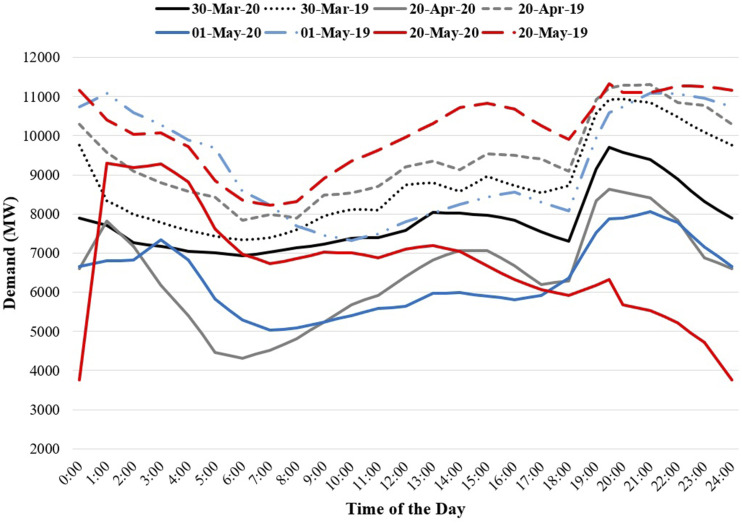
Four different days’ demand profiles during the lockdown period in 2020 compared with the same calendar days’ demand in 2019 (for interpretation of the references to color in this figure legend, the reader is referred to the Web version of this article).

To cross-check with individual daily average hourly demand profiles, four days were randomly selected during the lockdown time in 2020 and compared with the same calendar days’ demand in 2019. These demand profiles are illustrated in Fig. 9. It can be seen that at the beginning of the lockdown, that is, the last week of March 2020, the demand reduction was not significant. Later, in April and May, the national daily demand reduction was noticeable.

Electricity demand variations were reported in many recent studies, predominantly for the developed world. For example, about 12%, 20%, and 25% demand reductions were found in the UK, France, and Italy, respectively (Energy World, 2020). Gillingham *et al.* (2020) reported a 10% demand reduction in the USA. Similarly, there was a reduction of 14% of electricity demand due to this pandemic in ON, Canada (Abu-Rayash and Dincer, 2020). On the other hand, in developing countries such as India and Nepal, demand reductions were 19%–40% (Aruga *et al*., 2020 and Aggarwal, 2020) and 21%–28% (Nepali Times, 2020 and Bhushal, 2020), respectively.

Electricity consumption was 12 950 GWh in 2019, but this reduced to 11 098 GWh in 2020 during Bangladesh’s lockdown period, an ∼14% reduction. Similar reductions were also observed in many other countries; for example, about 10%–15% electricity consumption reduction occurred in Austria and Denmark during the lockdown period (EURELECTRIC, 2020). Electricity demand and consumption-related changes during the lockdown in many European countries can be found in EURELECTRIC (2020).

The analysis also explored the carbon intensity levels of nine different electricity generation zones in Bangladesh. In 2019, the most carbon-intensive zone was Dhaka as the average hourly CI range was found to be 145–159 gCO_2_*e*/kWh, followed by Sylhet (67–83 gCO_2_*e*/kWh), Cumilla (63–67 gCO_2_*e*/kWh), and Rajshahi (59–67 gCO_2_*e*/kWh). In contrast, the lowest carbon intensive zone was found to be Barisal (hourly average: 14–21 gCO_2_*e*/kWh), followed by Mymensingh (hourly average: 16–24 gCO_2_*e*/kWh). However, the hourly CI fluctuations were higher for Mymensingh than for Barisal.

Although the CI reduced for the Dhaka zone during the lockdown, it remains the most carbon-intensive zone. A recent study also found overall emission reduction in Dhaka due to the lockdown (Arora *et al*., 2020). For all other zones, the hourly average CI reduced, except Barisal, where a new coal-fired power plant came online in January 2020 with ultra-supercritical technology for emission control. However, the CI level increased significantly in this zone, a two-to three-fold increase in hourly average CI (42–50 gCO_2_*e*/kWh). The overall change in CI patterns for the nine zones and the national pattern is summarized in Table IV

**TABLE IV.** Carbon intensity changes in different zones with and without the lockdown.

	No pandemic, no lockdown	COVID-19 pandemic and
Zone	year: 2019	lockdown year: 2020
Barisal	Minor carbon peak in the evening.	The overall pattern has not changed, but hourly carbon intensity fluctuation increased significantly due to the addition of a coal-fired power plant.
Chattogram	Almost a flat carbon intensity pattern with a minor evening carbon peak.	Hourly CI fluctuations with a clearly visible evening carbon peak.
Cumilla	Almost a flat carbon intensity pattern with a minor day carbon peak.	Almost a flat carbon intensity pattern with no carbon peak.
Dhaka	Two clear carbon peaks: One at about 3 pm in the afternoon and another in the evening at 8 pm.	Two minor carbon peaks: One in the early morning and another in the afternoon.
Khulna	One clear evening carbon peak.	One clear evening carbon peak and hourly CI fluctuations were reduced.
Mymensingh	A midnight carbon peak and hourly CI fluctuations all through the day.	Morning and evening carbon peaks with hourly CI fluctuations all through the day.
Rajshahi	An evening peak and hourly CI fluctuations all through the day.	Two carbon peaks: One during the day and another in the evening with hourly CI fluctuations
Rangpur	One morning carbon peak.	One morning carbon peak and hourly CI fluctuations all through the day.
Sylhet	One morning peak and an evening “carbon valley.”	One day peak.
National	A clear carbon peak in the evening.	A clear carbon peak in the evening and hourly CI fluctuations all through the day.

**FIG. 10.**
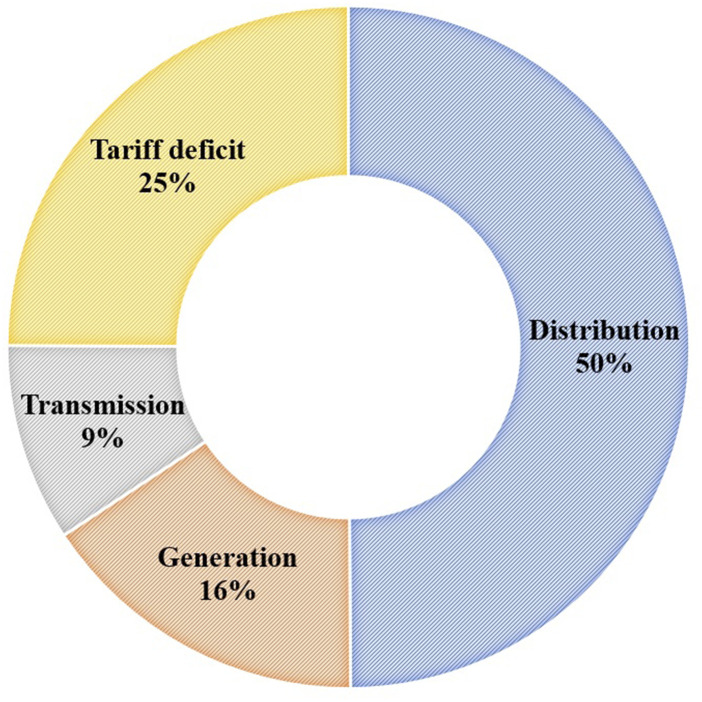
Power sector’s estimated loss from different sub-sectors due to the COVID-19 pandemic from March–December 2020 in Bangladesh [date source: Sajid (2020b)].

This coronavirus pandemic and the worldwide economic shocks are forcing governments worldwide, including Bangladesh, to assess the resilience of the power sector. Due to this crisis, it is expected that there would be a severe impact on the energy sector. For example, in terms of economic evaluation, the estimated loss in Bangladesh’s power sector due to the pandemic from March to December 2020 is expected to be BDT [Bangladeshi taka (Bangladeshi currency)] 354.07 × 10^9^, and the breakdowns are shown in Fig. 10. About half of the total loss was from the distribution sector, which is about BDT 176.66 × 10^9^, followed by a tariff deficit.

This analysis provides several insights into a fossil fuel-dominated electricity system. First, the COVID-19 pandemic reduced emission levels from electricity generation compared to the same period in the previous year. However, this type of reduction is due to crisis management-related energy use and on a very short-term basis. Although this may be true, it shows how far it is possible to control emissions from a fossil-fueled electricity generation system by suspending all types of industrial and commercial activities. This might help set targets for the electricity sector’s emission control to achieve sustainable development goal-7 (UN, 2015).

Second, the electricity demand reduced due to the shutdown of many industrial and commercial activities. Correspondingly, consumption at residences increased, and the electricity sector of Bangladesh was dominated by the residential consumer. To reduce electricity demand along with emissions on a long-term basis, separate initiatives should thus be considered. For instance, demand-side management (DSM) in every sector (e.g., residential and commercial) could be a valuable option for achieving this aim.

Third, Bangladesh’s electricity generation expansion plan is dominated by fossil fuels (Khan, 2018), and very few options are available to integrate renewable sources due to resource unavailability. Thus, to reduce demand along with DSM schemes, efficiency improvement in home appliances might be an effective option as more than 50% of consumers are from residences.

Fourth, this analysis used actual power plant-specific efficiency for generation emission factor calculations. Thus, it provides more accurate carbon intensity levels for a zone and the country. For example, in several previous studies, average efficiency was considered for different types of power plants in Bangladesh, and they found that the hourly average CI varied between 660 and 685 gCO_2_*e*/kWh for the year 2015 (Khan, 2018). Another study found that “the median daily time-varying carbon intensity fluctuated between 647 and 695 gCO2-*e*/kWh” across all seasons in Bangladesh in 2015 (Khan, 2019d). In contrast, this analysis revealed that national carbon intensity variations were 496-530 gCO_2_*e*/kWh in 2019 but 488–514 gCO_2_*e*/kWh in 2020 during the lockdown period. This implies that the use of actual efficiencies for individual power plants provides more accurate carbon intensity measures for a country.

Apart from these, there is a huge challenge after the pandemic: plastic waste management, mainly plastic used for personal protection items in the medical sector. One of the very common practices in managing this waste is the waste-to-energy conversion through incineration, and most of the developed nations would be following this practice. Although these plastic wastes have worthwhile calorific value for electricity generation, they emit GHGs (Klemeš *et al*., 2020).

Based on the results obtained here, it is clear that even with the country-wide closure of industrial and commercial activities for more than two months, the reduction in GHGs from the electricity sector is not significant (16% only), compared to the previous year. This indicates that to reduce GHG emissions from a fossil fuel electricity sector such as Bangladesh to ensure sustainable development, several policies need to be implemented. These policies could also be adopted by countries with similar electricity systems and where the integration of new renewable sources is not feasible due to resource limitations. Overall, if demand can be reduced, related GHG emissions from this fossil fuel sector can also be minimized.

## V. CONCLUSION AND POLICY IMPLICATIONS

Globally, the COVID-19 pandemic has affected the power sector in many ways. For instance, demand was reduced, triggering unprecedented instability in energy markets. This can be seen from recent oil price falls in the international market. However, in some countries, residential electricity consumption increased. On the other hand, as demand decreased, GHG emissions also declined in a fossil fuel-dominated electricity sector, and consequently, environmental pollution was reduced.

Notably, the pandemic hindered global economic growth as electrical energy is the driving force for this growth, predominantly in emerging economies, and Bangladesh is no exception. Similar to many other emerging economies, Bangladesh’s electricity sector has also been affected by the pandemic. This study’s objective was to assess the impact of the pandemic on the electricity sector of Bangladesh, which has a fossil fuel-dominated electricity generation system. The findings from this study could also be applied to countries with similar electricity generation systems.

The analysis revealed that during the country-wide lockdown, electricity demand varied from one zone to another. Significant demand reduction was observed for Dhaka and Chattogram during the lockdown period compared to other zones. For other zones, the daily demand variations were marginal. In terms of national hourly demand variation, the maximum and minimum demand reductions were found in the afternoon (5–6 pm) and evening (7–8 pm), network peak hours.

Carbon intensity also varied from one zone to another. For absolute zonal emission, the maximum emission reduction was found for Dhaka (33.81%) followed by Chattogram (28.81%), with a minimum in Rangpur of 9.72%. Notably, emissions increased by 124% in Barisal due to the addition of a new coal-fired power plant in this zone. Time-varying carbon intensity also varied from one zone to another. Due to the lockdown, carbon peaks also shifted from one hour to another. For example, a morning carbon peak in Sylhet moved to 11 am and became a day carbon peak.

This study provided detailed insights into Bangladesh’s electricity sector changes due to the COVID-19 crisis and recommended policy implications toward sustainable development in the energy sector. The study also has a few limitations. First, this analysis has not considered indirect emissions from renewable sources such as the life cycle emissions from solar and hydropower plants. Second, the oil-fired power plants in Bangladesh use high-speed diesel (HSD) and heavy fuel oil (HFO) as their primary fuels. However, the emission factor was considered for distillate fuel oil No. 2, which has similar characteristics to HSD and HFO but is not the same. Thus, the carbon intensity for the oil-fired power plant might vary slightly from the results obtained.

Similarly, the emission factor for sub-bituminous coal was taken into account for this analysis for the new coal-fired power plant in Barisal. Predominantly, this coal is imported from different countries such as Indonesia and Australia, and based on the coal quality, the emission factor might vary marginally. Although true, the carbon intensity variations due to marginal emission factor changes would be insignificant. Apart from this, the imported electricity from India was not considered for the analysis.

Based on this analysis, the following policy implications could be applicable for the developing world with fossil fuel dominated electricity systems:

(i) Effective DSM schemes at residences need to be deployed for electricity demand reduction. Previous studies have found that different DSM schemes need to be employed at different stages. For instance, energy-saving behaviors could be an effective primary method that could effectively reduce residential demand by about 21.9% (Khan, 2019a). The next DSM scheme after energy-saving behavior could be technology involvement in DSM. However, to manage residential demand effectively with technology, the factors that dominate electricity demand at residences (Khan, 2019b) need to be identified through appropriate methods (Khan *et al*., 2019).
(ii) Efficiency improvement in electrical appliances is another means of reducing electricity demand, not only for the industrial and commercial sectors but also in the domestic sector. However, such an improvement does not have a substantial impact on peak demand reduction (Borg and Kelly, 2011). One study found that home appliances' efficiency improvement could reduce energy consumption by about 11%–38% (Khan, 2019a).
(iii) More renewable generation options for the electricity generation system need to be explored. For example, Bangladesh does not have many other renewable generation options, except solar. However, due to the scarcity of land, it is difficult to achieve a substantial effect through solar generation in the country. There are also other options that require further research, which could be used as potential renewable sources to generate electricity, such as waste to energy schemes (Khan and Kabir, 2020 and Khan, 2019c).
(iv) Cross-border electricity trading might be another option to reduce demand in a fossil fuel-dominated electricity generation system, along with the security of supply (Kumar Singh, 2013). For instance, Bhutan, Nepal, and India have a total estimated capacity of 263 GW hydropower potential (Khan, 2020a). If these renewable energy potentials can be utilized through cross-border energy trading, a large amount of GHGs from the electricity sectors of Bangladesh and India could be saved. At the same time, this would underpin economic growth and other benefits in this region (Alam *et al*., 2019).
(v) As Bangladesh is a least developed country, research in the energy management and sustainability field is limited due to many constraints, such as inadequate funding. Thus, a lot of research needs to be conducted to identify potential solutions that could help reduce emissions from the electricity sector. One such area could be waste-to-green hydrogen generation and its application in the electricity generation sector (Khan, 2020b).

## Data Availability

The data that support the findings of this study are available from the corresponding author upon reasonable request.
